# A Simulated Visual Field Defect Impairs Temporal Processing: An Effect Not Modulated by Emotional Faces

**DOI:** 10.3390/vision9030079

**Published:** 2025-09-16

**Authors:** Mohammad Ahsan Khodami, Luca Battaglini

**Affiliations:** Department of General Psychology, University of Padua, 35131 Padova, Italy

**Keywords:** temporal processing, two-flash fusion, artificial scotoma, emotional faces, visual perception

## Abstract

Temporal processing is fundamental to visual perception, yet little is known about how it functions under compromised visual field conditions or whether emotional stimuli, as reported in the literature, can modulate it. This study investigated temporal resolution using a two-flash fusion paradigm with a static, semi-transparent overlay that degraded the right visual hemifield of opacity 0.60 and examined the potential modulatory effects of emotional faces. In Experiment 1, participants were asked to report if they perceived one or two flashes presented at either −6° (normal vision) or +6° (beneath a scotoma) across eight interstimulus intervals, ranging from 10 to 80 ms with a step size of 10 ms. Results showed significantly impaired temporal discrimination in the degraded vision condition, with elevated thresholds 52.29 ms vs. 34.78 ms and reduced accuracy, particularly at intermediate ISIs 30–60 ms. In Experiment 2, we introduced emotional faces before flash presentation to determine whether emotional content would differentially affect temporal processing. Our findings indicate that neither normal nor scotoma-impaired temporal processing was modulated by the specific emotional content (angry, happy, or neutral) of the facial primes.

## 1. Introduction

The human visual system must process information across both spatial and temporal dimensions to construct a coherent representation of the dynamic visual world [1,2,3,4,5]. Temporal resolution—the ability to correct perceive of a visual stimuli presented in rapid succession—is fundamental to many aspects of visual perception [6,7], including motion detection [8], reading [9,10], and object recognition [11,12]. One of the most well-established paradigms for examining temporal resolution in vision is the two-flash fusion task [7,13,14,15].

In the two-flash fusion paradigm (TFF), participants must discriminate between a single flash and two sequential flashes presented with varying interstimulus intervals (ISIs) [7,14]. The threshold at which two distinct flashes are perceived as a single event defines the temporal resolution limit of the visual system under specific conditions [7,14,15,16,17].

The TFF thresholds and similar functions under the flicker fusion paradigm have been linked to cognitive functions beyond basic visual processing. TFF performance has been associated with executive function across age groups, suggesting its potential as a predictor of cognitive abilities [18]. Additionally, TFF thresholds are affected by factors such as viewing conditions, arousal levels, and circadian rhythms [14,19]. Interestingly, spatial attention has been found to degrade temporal resolution as measured by TFF, contrary to its enhancing effect on spatial resolution [15]. These findings highlight the complex relationship between visual processing speed and higher-order cognitive functions, as well as the sensitivity of TFF to both state and trait variations in individuals [14].

Additionally, recent work by [16,20,21] has demonstrated that perceptual learning through visual training can enhance temporal processing as measured by the TFF. Perceptual learning based on temporal stimuli has been found to improve both temporal and spatial visual performance in amblyopic adults, with improvements in visual acuity, contrast sensitivity, and stereopsis [16]. These findings highlight the potential for perceptual learning in visual rehabilitation contexts.

Visual field defects profoundly affect visual perception and everyday functioning [22]. A scotoma is defined as a localized area of diminished or absent vision within the visual field, often surrounded by regions of intact vision [23,24,25]. These defects can arise from various pathologies affecting the visual pathway, including retinal disorders like age-related macular degeneration and neurological conditions such as stroke, causing hemianopia [24,26,27,28].

Hemianopia, resulting from damage to the post-chiasmatic visual pathway, presents a particularly challenging condition that affects approximately 30% of stroke survivors [27,29,30,31]. Individuals with hemianopia not only experience difficulties in spatial perception but often demonstrate impairments in temporal processing within their affected visual fields [29,30,31,32]. However, studying these temporal deficits directly in clinical populations presents methodological challenges due to the heterogeneity of lesion sites, comorbidities, and compensatory mechanisms that develop post-injury [33,34].

To model the perceptual consequences of visual field defects, researchers have often used “artificial scotoma” paradigms. A sophisticated approach involves a gaze-contingent display, where an eye-tracker is used to occlude a specific portion of the participant’s visual field, ensuring the “scotoma” remains stable regardless of where they look (e.g., [35]). This method directly simulates the experience of a retinal blind spot.

In the current study, we employ a simpler, non-contingent method (hereby we call it scotoma) to investigate how temporal processing is affected by a sustained degradation of the visual signal. We created a condition of impaired visibility by overlaying a static, semi-transparent mask on one half of the display. While this does not simulate the neurological condition of hemianopia directly, it allows for a controlled examination of how the visual system performs a fine temporal discrimination task when the incoming sensory information is noisy or attenuated. This approach allows us to probe the fundamental resilience of temporal processing mechanisms when faced with compromised input, a condition that shares features with, but is not identical to, clinical visual field loss.

Studies using artificial scotomas have revealed important insights into adaptation mechanisms [35,36]. For example, research by [36,37] demonstrated that artificial scotomas undergo perceptual filling-in, where the occluded region appears to be filled with the surrounding texture or pattern. This phenomenon suggests active neural compensation processes at work. More recent studies using functional MRI have shown that perceptual completion of artificial scotomas is associated with modulations of neural activity in early visual cortical areas, including V1 and V2, supporting theories of rapid visual plasticity [38].

Artificial scotoma paradigms have also been employed to investigate how individuals adapt their visual search strategies when faced with field defects. Work by [39] has shown that artificial scotomas affect not only what individuals can see but also how they program eye movements, with implications for understanding the functional adaptations required in actual visual field loss. As noted by [40] visual field defects may render oculomotor adaptation suboptimal, suggesting that visual deficits impact perception and the strategic deployment of visual attention.

Emotional processing represents another important dimension of visual perception [41,42], particularly in relation to social stimuli such as faces. Numerous studies have demonstrated that emotional content can modulate various aspects of visual processing, including attention allocation, perceptual sensitivity, and temporal dynamics [41,43,44]. Emotional faces, in particular, have been shown to capture attention rapidly and influence subsequent visual processing operations [43]. The neural mechanisms supporting emotional face processing involve a distributed network of brain regions [45,46,47]. According to the influential model proposed by [48] face processing engages both a core system and an extended system. Within this framework, the superior temporal sulcus is crucial for processing dynamic facial information, including emotional expressions and eye gaze [49].

Emotional content’s influence on visual perception’s temporal aspects has been investigated using various paradigms. For instance, emotional stimuli have been shown to modulate the attentional blink, temporal order judgments, and duration estimations [50,51,52]. These findings suggest that emotion can influence the temporal resolution of visual processing. Recently, it was demonstrated that categorical perception of facial expressions occurs as early as 160 ms following stimulus onset in occipitotemporal regions, with happiness decoded approximately 20 ms faster than fear. This highlights differential processing speeds for different emotions.

A significant area of relevance to the current investigation is the scarcity of studies directly examining the influence of emotional content on two-flash fusion thresholds or temporal integration processes in the context of compromised visual input, such as artificial scotomas. This signifies a gap in the understanding of the interaction between emotional and temporal processing in vision, particularly under conditions of visual field deficits. The present investigation aims to bridge these research domains by investigating the impact of artificial scotomas on temporal processing, as assessed through the two-flash fusion paradigm, and determining whether emotional faces exert a differential influence on this process. We conducted two complementary experiments precisely designed to systematically address these inquiries.

Experiment 1 established baseline effects by comparing two-flash fusion performance between normal vision (left visual field, −6°) and scotoma conditions (right visual field, +6°). We measured accuracy across multiple interstimulus intervals to characterize the temporal window within which the scotoma most strongly impacts flash discrimination.

In Experiment 2, we introduced emotional faces (angry, happy, and neutral) immediately before the flash detection task to determine whether emotional content would differentially affect temporal processing in normal versus scotoma conditions. This design allowed us to investigate potential interactions between emotional processing and temporal vision in the context of simulated visual field defects.

Our specific research questions were:

1. Compared to normal vision, does an artificial scotoma impair temporal processing as measured by the two-flash fusion paradigm?
2. If temporal processing is impaired under scotoma conditions, does this impairment manifest uniformly across all interstimulus intervals, or are there specific temporal windows where the effect is most pronounced?
3. Do emotional faces (angry, happy, neutral) modulate temporal processing in the two-flash fusion task, and does this modulation differ between normal vision and artificial scotoma conditions?
4. Are reaction times affected by the presence of an artificial scotoma, emotional content, or their interaction?

By addressing these questions, this study aims to enhance our understanding of how visual field disruptions affect temporal processing and whether emotional content can influence these processes.

## 2. Materials and Methods

### 2.1. Stimuli and Apparatus

The experiment was conducted in a controlled, dimly lit room. Participants were positioned at a distance of 57 cm from the screen and used a chin rest for stabilization. The experimental design was executed using Psychopy Coder [53,54]. The stimuli were displayed on 2560 × 1440 pixels monitor with a refresh rate of 240 Hz with a base 350 cd/m^2^ luminance, due to the need to replicate the experiment on the current and accurate refresh rate, achieving our needed ISI, we have set the monitor to a 100 Hz refresh rate and set the average luminance to 150 cd/m^2^. The displays featured a medium gray background (RGB: 128, 128, 128) with a central fixation cross (RGB: 255, 255, 255). To create a condition of degraded visual input, a semi-transparent black rectangle (0.60 opacity) resulting in a background color of RGB (51, 51, 51) in that region was overlaid on the entire right half of the display and a central white fixation cross spanning 1° of visual angle was present while our use of an eye-tracker ensured central fixation at the start of each trial. The flash stimulus comprised a sinusoidal grating with a Gaussian envelope (a Gabor patch) spanning 2 degrees of visual angle. The grating exhibited a spatial frequency of 1 cycle per degree and a 30% contrast relative to its immediate background. Flash stimuli were presented at one of two positions: −6° (left of fixation, in the normal visual field) or +6° (right of fixation, in the area obscured by the scotoma) (see Supplementary Materials).

For Experiment 2, emotional face stimuli were sourced from a standardized database and presented centrally on the screen. Faces were 450 × 450 pixels in size and displayed one of three emotional expressions: happy, angry, or neutral. Based on data from [55]. The happy faces had a mean valence of 4.88, the neutral faces had a mean of 3.92, and the angry faces had a mean of 2.89, and both male and female face images were used to add variability and generalizability.

### 2.2. Experiment 1: Two-Flash Fusion with Scotoma

#### 2.2.1. Participants

Based on [55] a sample of 21 participant would be enough for this study, but a total of 25 (13 Female, Mean Age 30.53 ± 4.66; 88% were right handed) participants with normal or corrected-to-normal vision completed the experiment. Demographic information was collected before testing, including age, handedness, vision problems, and psychological conditions.

#### 2.2.2. Experimental Design

Experiment 1 employed a within-subjects design with two independent variables: position (−6° or +6° from fixation) and interstimulus interval (ISI: 10, 20, 30, 40, 50, 60, 70, or 80 ms) [56]. The dependent variables were discrimination accuracy and reaction time. The experiment consisted of 400 trials (50 trials per ISI, split equally between the two positions), presented in a randomized order. Trials were randomly assigned.

#### 2.2.3. Procedure

**Figure 1:**
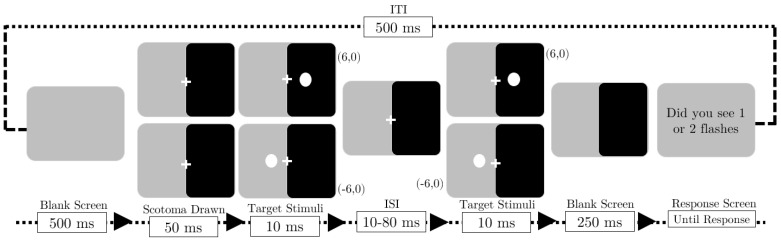
Schematic of the trials in Experiment 1, Each trial began with a central fixation cross displayed for 500 ms, followed by the presentation of the scotoma that covered the right half of the screen 50 ms before the drawing of flashes. The first flash appeared at either −6° (**left**) or +6° (**right**) for 10 ms, and this was followed by a variable inter-stimulus interval (ISI) ranging from 10 to 80 ms, where only the overlay and fixation cross were visible. The second flash then appeared at the exact location as the first for another 10 ms, followed by an elapsed period of 250 ms in which only the scotoma remained visible. Finally, a response prompt appeared, asking, “Did you see one or two flashes?” Participants were instructed to press ‘z’ for one flash or ‘m’ for two flashes.

Each trial followed this sequence as depicted in Figure 1; Each trial began with a central fixation cross displayed for 500 ms, after which the scotoma was presented covering the right half of the screen from 50 ms before showing flashes. The first flash then appeared at either −6° (left) or +6° (right) for 10 ms, followed by a variable ISI period (10–80 ms), The second flash appeared at the exact location as the first flash for 10 ms, after which another 250 ms blank screen. A response prompt then appeared asking “Did you see one or two flashes?” with instructions to press ‘z’ for one flash or ‘m’ for two flashes. Upon receiving the participant’s response, a 500 ms inter-trial interval preceded the beginning of the subsequent trial.

Participants were instructed to maintain fixation on the central cross throughout each trial. To control this, we checked their fixation using an EyeLink 1000 Plus tower with a drift of a circle with a diameter of 2 visual degrees from the center, 1 degree per side, and up and down. The entire experiment took approximately 30–40 min to complete.

### 2.3. Experiment 2: Influence of Emotional Faces on Two-Flash Fusion Under Artificial Scotoma Conditions

#### 2.3.1. Participants

The participants in this experiment were the same as those in Experiment 1.

#### 2.3.2. Experimental Design

Experiment 2 employed a within-subjects design with three independent variables: position (−6° or +6° from fixation), interstimulus interval (ISI: 10, 20, 30, 40, 50, 60, 70, or 80 ms), and emotional face type (happy, angry, neutral). Again, the dependent variables were discrimination accuracy and reaction time. The experiment consisted of 400 trials, with the same distribution as Experiment 1 but with emotional face conditions randomly assigned across trials.

#### 2.3.3. Procedure

**Figure 2:**
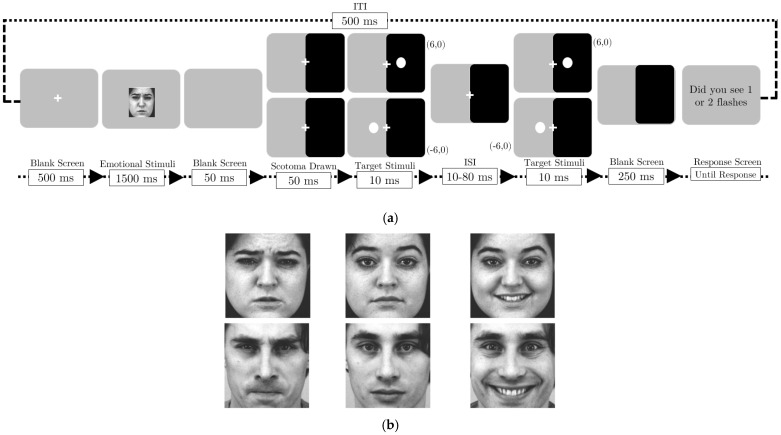
Panel (**a**) schematic illustration of Experiment. The trial sequence was identical to Experiment 1, with the addition of a preceding emotional prime. Following the initial fixation, an emotional face was presented centrally for 1500 ms, followed by a 50 ms blank screen. The dark overlay was then presented on the right hemifield for 50 ms, after which the two-flash fusion task commenced as described in Figure 1. Panel (**b**) illustrates the imagery employed in this study for the experiment [55].

The procedure in the experiment was close to Experiment 1, with an extra stimulus shown (the emotional faces), as depicted in Figure 2, Each trial in Experiment 2 began with a central fixation cross displayed for 500 ms without the scotoma present. An emotional face (happy, angry, or neutral) then appeared centrally for 1500 ms, followed by a blank screen for 50 ms, both presented without the scotoma. The artificial scotoma was then introduced, covering the right half of the screen, after which the first flash appeared at −6° (left) or +6° (right) for 10 ms. A variable ISI period (10–80 ms) followed by a second flash at the exact location for 10 ms, a post-flash delay of 250 ms, and a blank screen. A response prompt then appeared asking “Did you see one or two flashes?” with instructions to press ‘z’ for one flash or ‘m’ for two flashes. After the participant responded, a 500 ms inter-trial interval (ITI) preceded the beginning of the subsequent trial.

The key difference from Experiment 1 was the presentation of emotional faces before each trial’s flash sequence. Participants were instructed to view the faces but were told that their task remained the same: to report whether they perceived one or two flashes. The scotoma was only introduced after the emotional face presentation, immediately before the first flash stimulus. As in Experiment 1, the session took approximately 40–45 min.

### 2.4. Data Analysis

All data were analyzed in Python Software (Python 3.12.10), using the Jupyter notebook environment V2025.8.0. Data were analyzed using statsmodels V0.14.4 [57], scipy V1.16.1 [58] and plots were created using Matplotlib V3.10.0 [59] libraries. Before processing, a pre-check was performed to check if any missing data had been recorded.

For both experiments, accuracy was calculated as the proportion of correct responses (i.e., correctly reporting two flashes) for each combination of conditions. Error rates were also calculated, where an error was defined as reporting one flash when two flashes were presented. Reaction times were measured from the onset of the response prompt to the participant’s keypress.

Primary analyses examined the effects of position and ISI on flash detection accuracy (Experiment 1) and the additional influence of emotional faces on this process in Experiment 2. As accuracy is a binary measure, this data was analyzed using Generalized Linear Mixed-Effects Models (GLMMs). The continuous reaction time data, however, were analyzed using repeated measures ANOVAs. Additionally, psychometric functions were fitted to the accuracy data to determine discrimination thresholds for each condition.

## 3. Results

### 3.1. Experiment 1: Two-Flash Fusion Detection with Artificial Scotoma

#### 3.1.1. Accuracy Analysis

To test the effects of visual field degradation on temporal resolution, we analyzed the trial-level accuracy data using a Generalized Linear Mixed-Effects Model (GLMM) with position, ISI, and their interaction as fixed effects and participant as a random effect. The analysis revealed a significant main effect of Position (χ^2^(1) = 897.27, *p* < 0.001), with lower accuracy in the scotoma condition compared to the normal vision condition. A significant main effect of ISI was also observed (χ^2^(7) = 5562.09, *p* < 0.001), with detection accuracy improving as the interstimulus interval increased. Critically, there was a significant Position × ISI interaction (χ^2^(7) = 247.79, *p* < 0.001), indicating that the effect of ISI on accuracy differed between the normal vision and scotoma conditions. Specifically, the performance gap between conditions was largest at intermediate ISIs (30–60 ms) and minimal at both the shortest (10–20 ms) and longest (80 ms) ISIs.

**Figure 3:**
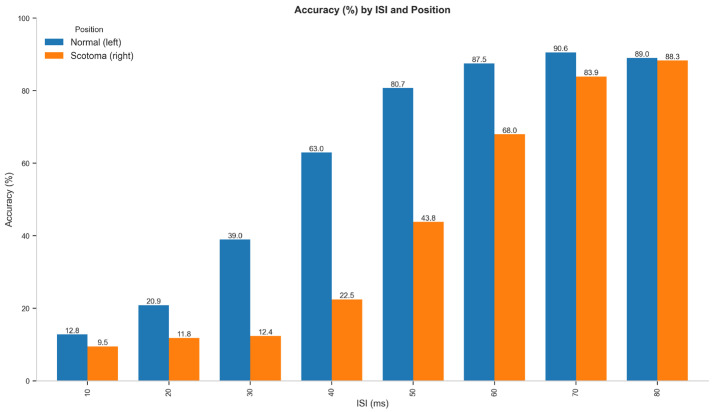
The percentage of accuracy in both conditions across different ISIs shows that there is no substantial difference in the shorter ISI, including 10 ms, making it challenging to analyze rather than the position. Additionally, at 80 ms, the longer ISI can lead to better discrimination between one or two flashes, regardless of the position.

**Figure 4:**
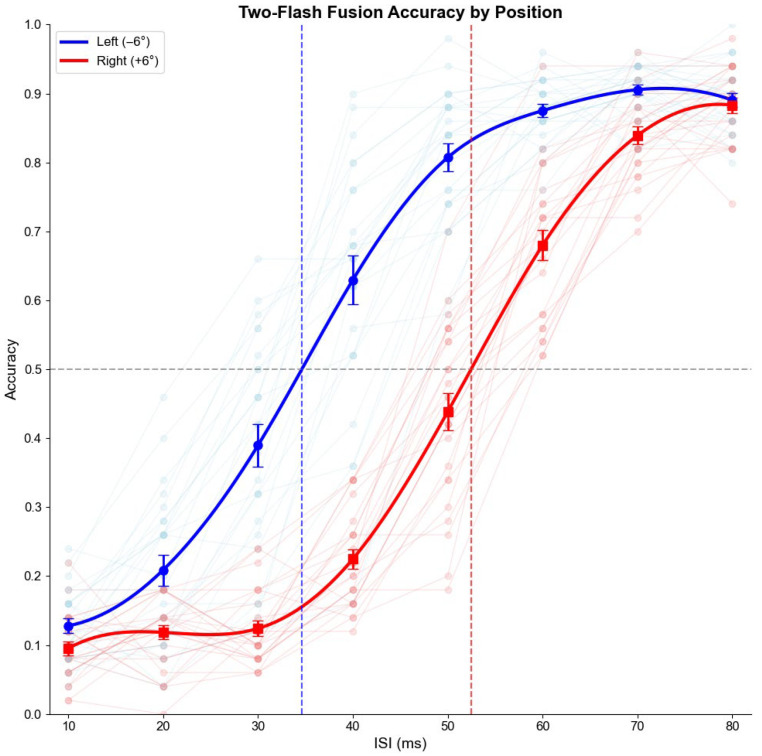
Psychometric functions for two-flash fusion accuracy as a function of interstimulus interval (ISI) in Experiment 1. Accuracy is plotted separately for the normal vision condition (**Left**, −6°, blue) and the degraded vision condition (**Right**, +6°, red). Individual participant data are shown as faint lines, and group means with standard errors are shown as filled symbols with error bars. Solid curves represent fitted psychometric functions. Dashed horizontal line indicates the 50% accuracy threshold, and vertical dashed lines mark the group-level thresholds for each condition (Normal ≈ 35 ms; Scotoma ≈ 52 ms). The results show impaired temporal resolution in the scotoma condition, reflected in a rightward shift of the psychometric function.

Table 1 presents the accuracy rates by ISI and position for Experiment 1. Figure 3 shows the plot bar, and Figure 4 presents the psychometric function of this experiment. The data shows that at the shortest ISI (10 ms), accuracy was low in both conditions (normal: 12.8%, scotoma: 9.5%), suggesting that the flashes were too rapid to be distinguished regardless of position. At intermediate ISIs, the difference became substantial, with the most significant disparity occurring at 40 ms (normal: 63.0%, scotoma: 22.5%) and 50 ms (normal: 80.7%, scotoma: 43.8%). As ISI increased to 80 ms, the performance gap narrowed considerably (normal: 89.0%, scotoma: 88.3%), indicating that with sufficient temporal separation, performance in the scotoma condition approached normal vision.

Figure 4 shows the psychometric function for both conditions across ISIs. To achieve a minimum accuracy of 50%, approximately 35 ms is needed for the left (normal condition), whereas for scotoma, this duration is around 52 ms, indicating the impact of scotoma on the process.

Paired *t*-tests at each ISI level confirmed significant differences between positions for ISIs of 20–70 ms (all *p* < 0.001 after Bonferroni correction), but not for the shortest (10 ms) and longest (80 ms) ISIs. The largest effect sizes were observed at 40 ms (Cohen’s *d* = 3.03) and 50 ms (Cohen’s *d* = 3.13). A linear mixed-effects model with participant as a random effect further confirmed these findings, showing significant interaction effects between position and ISI for the 30–60 ms intervals (all *p* < 0.001).

#### 3.1.2. Threshold Analysis

As shown in Figure 4 and Table 2, psychometric curves were fitted to determine the ISI threshold at which participants achieved 50% accuracy in detecting two flashes. In the normal vision condition (−6°), the mean threshold was 34.78 ms (95% CI: 32.42–37.14 ms). In contrast, the artificial scotoma condition (+6°) showed a significantly higher threshold of 52.29 ms (95% CI: 50.00–54.59 ms). Individual participant thresholds showed the same pattern, with mean thresholds of 34.59 ms (SE = 1.17) for the normal condition and 52.41 ms (SE = 0.75) for the scotoma condition.

Paired *t*-tests comparing individual thresholds between positions confirmed this difference was highly significant (*t*(24) = −13.71, *p* < 0.001) with a large effect size (Cohen’s *d* = −3.62). The elevated threshold in the scotoma condition indicates impaired temporal processing, suggesting that the artificial scotoma significantly disrupts the ability to discriminate between single and double flashes.

#### 3.1.3. Reaction Time Analysis

Reaction times also differed significantly between positions (*t* = −32.09, *p* < 0.001), with responses being slower in the scotoma condition (616.24 ms) compared to the normal condition (579.84 ms). This difference of approximately 36 ms suggests that processing visual information through an artificial scotoma reduces accuracy and increases processing time. Additionally, reaction times were significantly longer for incorrect responses (642.05 ms) compared to correct responses (556.57 ms; *t* = −85.90, *p* < 0.001), indicating that uncertainty in perception was associated with delayed responses. This pattern is consistent with the idea that when participants struggle to distinguish between one and two flashes, their decision-making process takes longer, regardless of the visual field position.

### 3.2. Experiment 2: Effect of Emotional Faces on Two-Flash Fusion

#### 3.2.1. Accuracy Analysis

Experiment 2 investigated whether emotional faces (Angry, Happy, Neutral) affected the two-flash fusion effect in both standard and scotoma conditions. Similarly to Experiment 1, we observed a significant main effect of position across all ISI levels, with poorer performance in the scotoma condition. Table 3 presents the overall accuracy rates by position and emotion. The data reveal that while the position effect remained robust (regular: ~60.7%, scotoma: ~41.7%), each position had minimal variation across emotional conditions.

**Figure 5:**
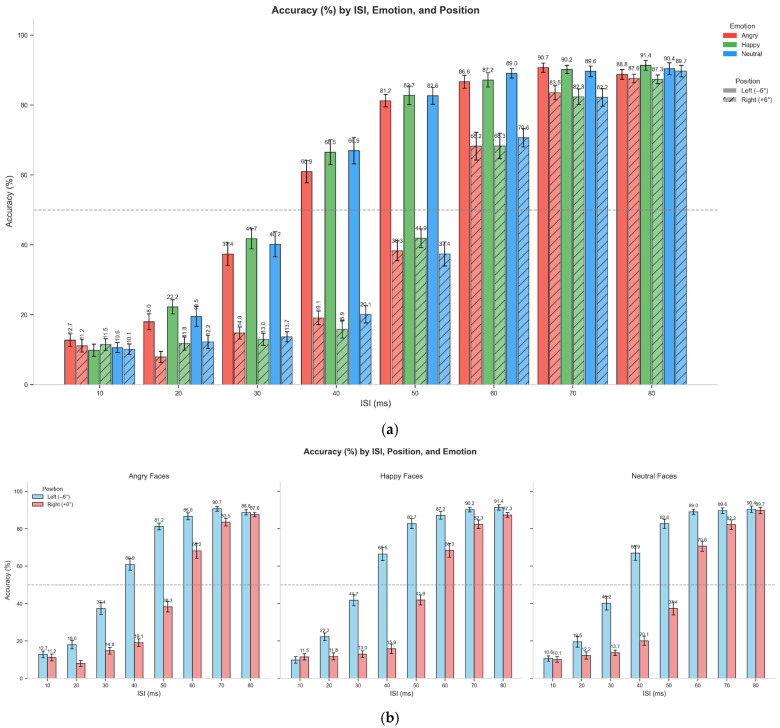
Panel (**a**) shows accuracy (%) in the two-flash fusion task as a function of interstimulus interval (ISI), separated by both emotion (angry, happy, neutral) and position (normal vision, −6°, solid bars; degraded vision, +6°, hatched bars). Group means with standard errors are displayed for each condition, with numerical values above bars indicating accuracy percentages. Across emotions. Panel (**b**) shows accuracy (%) in the two-flash fusion task across interstimulus intervals (ISIs), separated by position (normal vision, −6°, blue; degraded vision, +6°, red) and emotional prime (angry, happy, neutral). Each subplot illustrates one emotional condition, with mean accuracy and standard errors displayed for each ISI.

Figure 5 at the top shows the overall accuracy by ISIs and position across different emotions, while the bottom displays the demonstrated accuracy by position and emotion separately.

To test these observations, we analyzed the accuracy data using a Generalized Linear Mixed-Effects Model (GLMM). We employed a model including all main effects and the key two-way interactions (Position × ISI and Position × Emotion). The analysis confirmed significant effects of Position (χ^2^(1) = 980.92, *p* < 0.001), ISI (χ^2^(7) = 5566.17, *p* < 0.001), and a significant Position × ISI interaction (χ^2^(7) = 349.56, *p* < 0.001).

Crucially, there was no significant main effect of Emotion (χ^2^(2) = 3.42, *p* = 0.18) and no significant Position × Emotion interaction (χ^2^(2) = 2.53, *p* = 0.28). These results confirm that emotional primes did not significantly modulate temporal processing in either the normal or the degraded visual field.

#### 3.2.2. Threshold Analysis

**Figure 6:**
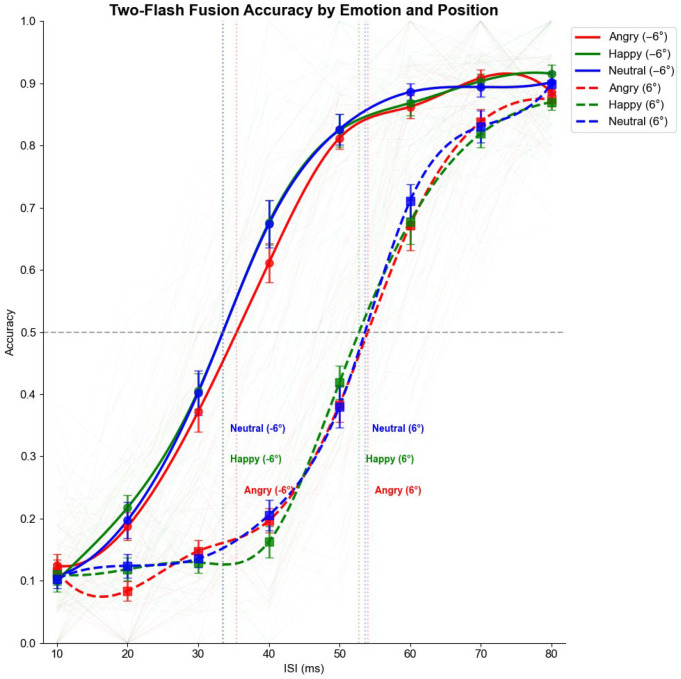
Psychometric functions for two-flash fusion accuracy as a function of interstimulus interval (ISI) in Experiment 2, plotted separately by emotion (angry, happy, neutral) and position (normal vision, −6°, solid lines; degraded vision, +6°, dashed lines). Individual participant data are shown as faint lines, while group means with standard errors are displayed as symbols with error bars. Vertical dotted lines indicate group-level thresholds for each condition. Accuracy increased with ISI across all conditions, and although performance was consistently impaired in the degraded (scotoma) hemifield relative to the normal hemifield, emotional primes (angry, happy, neutral) did not systematically shift thresholds or modulate accuracy.

Figure 6 displays the psychometric function from experiment 2, illustrating that the threshold for emotion priming is the same across both conditions.

Threshold analysis for Experiment 2, as depicted in Figure 6, revealed that the position effect remained consistent across emotional conditions, as shown in Table 4. For the normal (left) position, thresholds were 35.58 ms (95% CI: 33.04–38.10 ms) for angry faces, 33.80 ms (95% CI: 31.47–36.12 ms) for happy faces, and 33.95 ms (95% CI: 31.40–36.51 ms) for neutral faces. For the scotoma (right) position, thresholds were 53.51 ms (95% CI: 50.72–56.31 ms) for angry faces, 53.50 ms (95% CI: 50.40–56.61 ms) for happy faces, and 52.86 ms (95% CI: 49.84–55.88 ms) for neutral faces.

Individual threshold analysis using repeated measures ANOVA confirmed the strong main effect of position (*F*(1, 24) = 417.78, *p* < 0.001, η^2^*p* = 0.77) but showed no significant main effect of emotion (*F*(2, 48) = 1.64, *p* = 0.20) or position × emotion interaction (*F*(2, 48) = 0.65, *p* = 0.53). This suggests that emotional faces did not differentially affect the temporal processing thresholds in either the normal or scotoma conditions.

#### 3.2.3. Reaction Time Analysis

Analysis of reaction times showed a significant main effect of position (*F*(1, 24) = 311.80, *p* < 0.001, η^2^*p* = 0.72), no significant main effect of emotion (*F*(2, 48) = 0.27, *p* = 0.76), and no significant position × emotion interaction (*F*(2, 48) = 0.18, *p* = 0.83). As shown in Table 5, mean reaction times were consistently slower in the scotoma condition (~616 ms) compared to the normal condition (~579 ms) across all emotion conditions, with remarkably similar patterns across emotional contexts.

### 3.3. Comparison Between Experiments and Conclusions

Both experiments demonstrated consistent findings regarding the effect of artificial scotoma on two-flash fusion detection. The artificial scotoma consistently impaired performance, with particularly large differences at intermediate ISIs (30–60 ms). The scotoma condition showed significantly higher thresholds (~52 ms) compared to the normal condition (~34 ms) in both experiments, representing an approximately 50% increase in the time needed to distinguish between single and double flashes. This temporal processing impairment remained stable across both experiments.

Experiment 2 revealed that emotional faces did not significantly modulate the two-flash fusion effect in either normal or scotoma conditions. The thresholds and accuracy patterns remained remarkably stable across emotional conditions (angry, happy, and neutral), suggesting that the temporal processing impairment caused by the artificial scotoma is robust to emotional context. Both experiments showed consistently slower reaction times in the scotoma condition, independent of emotional context in Experiment 2, with a consistent difference of approximately 36–37 ms.

These findings demonstrate that scotoma significantly impairs temporal processing in the two-flash fusion paradigm, as evidenced by reduced accuracy, elevated thresholds, and slower reaction times. This effect is robust and appears to be immune to emotional modulation. The consistency of these results suggests that the artificial scotoma paradigm provides a reliable model for studying temporal processing impairments in the visual system, which may have implications for understanding conditions such as hemianopia, where similar processing deficits occur. The emotional immunity of this effect further suggests that the temporal processing disruption operates at a fundamental sensory level rather than being susceptible to higher-level emotional influences.

## 4. Discussion

The present study aimed to investigate temporal processing under degraded visual input conditions and to examine whether emotional faces modulate this processing. Our findings provide several important insights into the nature of temporal vision in compromised visual fields and the interaction between emotional and temporal processing.

Our findings from Experiment 1 consistently demonstrated that presenting scotoma significantly impairs temporal discrimination in the two-flash fusion paradigm. The accuracy rates were markedly lower and thresholds substantially higher when flashes were presented in the scotoma region (+6°) compared to the normal visual field (−6°). The threshold for the normal visual field (34.78 ms) aligned well with previous research on temporal processing in intact peripheral vision, closely matching the values reported by Neri and Levi [60], who found thresholds between 30 and 40 ms at similar eccentricities. In contrast, the scotoma condition yielded a substantially elevated threshold (52.29 ms), representing approximately a 50% increase in the time needed to resolve sequential stimuli.

This pattern of impairment was most pronounced at intermediate ISIs (30–60 ms), with performance differences diminishing at both the shortest (10–20 ms) and longest ISIs (80 ms). This non-uniform effect across temporal intervals suggests that the artificial scotoma does not simply reduce stimulus visibility uniformly but rather interacts with specific temporal processing mechanisms. This finding aligns with [36] research showing that perceptual completion processes for artificial scotomas are time-dependent and not instantaneous, supporting the notion that temporal dynamics are fundamentally altered under scotoma conditions.

The elevated thresholds observed in our scotoma condition mirror those reported in clinical populations with visual field defects. For instance, [32,61] found significantly higher two-flash fusion thresholds in the affected visual fields of hemianopia patients compared to their intact fields.

The convergence of performance at very short ISIs (10–20 ms) likely reflects floor effects, as these intervals are too brief for temporal discrimination regardless of scotoma presence, consistent with the temporal limits of visual processing identified by [62]. Conversely, the convergence at the longest ISI (80 ms) suggests that with sufficient temporal separation, the scotoma’s impact on temporal discrimination diminishes. This aligns with [41]. Neuroimaging findings show that neural responses to stimuli within artificial scotomas can approach normal levels when presentation parameters are optimized.

The reaction time data further support the notion that processing visual information through an artificial scotoma imposes additional cognitive demands. Participants responded significantly slower (by approximately 36 ms) when discriminating flashes in the scotoma condition, consistent with the findings of [39] who reported increased processing time for visual tasks under artificial scotoma conditions. This suggests that degraded visual input requires more extensive processing or introduces greater uncertainty in perceptual decision-making, as proposed in [40] model of visual search under scotoma conditions.

Contrary to our initial hypothesis based on the literature on emotional influences on visual perception [51,52,55,63], Experiment 2 revealed no significant modulation of temporal processing by emotional faces. The presentation of angry, happy, or neutral faces prior to the flash discrimination task did not differentially affect performance in normal or scotoma conditions. The absence of emotional effects was consistent across all ISIs and was observed for accuracy and reaction time measures.

This null result contrasts with previous findings by [44], who reported that emotional stimuli, particularly threatening faces, can enhance visual processing. Similarly, our findings differ from [55,64] demonstration of rapid categorical perception of emotional expressions with differential processing speeds for different emotions. Several interpretations of this discrepancy warrant consideration.

First, the temporal parameters of our experimental design may have limited the potential for emotional modulation. The 1500 ms face presentation followed by a 50 ms blank interval and the flash sequence may have created sufficient temporal separation to prevent effective emotional priming. Reference [46] has shown that emotional effects on visual processing are most pronounced within specific temporal windows, typically around 100–300 ms following emotional stimulus onset. Our paradigm’s timing may have fallen outside this optimal window, allowing the emotional activation to dissipate before the critical flash discrimination task.

Second, the scotoma may have imposed such strong constraints on visual processing that any potential emotional modulation effects were overshadowed. When the visual system is challenged by degraded input, as in the scotoma condition, it may prioritize basic sensory processing over affective influences. This interpretation aligns with [36] biased competition model of attention, where limited processing resources are allocated based on task demands and stimulus salience. The demanding nature of perceiving flashes under the scotoma condition likely consumed attentional resources that might otherwise have been influenced by emotional content.

Third, the mechanisms underlying two-flash fusion primarily involve early visual processing stages that may be relatively immune to emotional modulation. While ref. [65] have demonstrated that emotional faces can modulate early ERP components like P1 and N170, these modulations may not directly influence temporal resolution capacities. This interpretation suggests a dissociation between emotional processing pathways and the specific temporal processing mechanisms assessed in our two-flash fusion paradigm, consistent with the segregation of processing streams proposed in [46,47,48] model of face perception.

Finally, methodological factors related to spatial attention might have diminished the potential emotional effects. Ref. [66] showed that emotional processing effects are most robust for centrally presented stimuli and may weaken for peripheral stimuli. The spatial separation between the centrally presented faces and the peripherally presented flashes in our paradigm may have reduced any emotional transfer effects, particularly given the constraints on spatial attention documented by [39] under artificial scotoma conditions.

Our findings contribute to theoretical models of visual perception in several ways. First, they provide empirical support for the notion that temporal processing is not uniform across the visual field and can be significantly affected by visual defects. This extends [17] work on flicker fusion by demonstrating that temporal thresholds depend not only on stimulus properties but also on the integrity of the visual field. These results necessitate refinements to models of visual processing that must account for how the visual system handles degraded input.

Second, the differential effects of the artificial scotoma across various ISIs suggest that temporal processing involves multiple mechanisms with different susceptibilities to visual degradation. This aligns with [67] findings showing that different temporal processing mechanisms can be selectively enhanced through training, supporting the notion that temporal vision comprises dissociable components rather than a unitary system. Our results particularly complement the hierarchical models of visual processing, where higher temporal frequencies engage different neural mechanisms than lower frequencies [62].

Third, the absence of emotional modulation effects in our paradigm suggests potential boundaries for emotion-perception interactions. While refs. [50,52] have shown that emotions can influence various aspects of temporal perception, including judgments of duration. Our results suggest that such influences may not extend to all forms of temporal discrimination, particularly under challenging perceptual conditions. This finding helps delineate the scope of emotional influences on perception and suggests that the robustness of emotional effects may depend on the specific perceptual process being assessed.

From a clinical perspective, our findings have important implications for understanding and addressing the challenges faced by individuals with actual visual field defects. The elevated temporal thresholds observed in the artificial scotoma condition suggest that rehabilitation approaches for patients with hemianopia should consider temporal processing deficits in addition to spatial ones. This aligns with [68] recommendations that effective rehabilitation programs must address the multifaceted nature of visual deficits following brain injury.

The specific pattern of results—showing maximal impairment at intermediate ISIs—suggests that temporal parameters should be carefully considered in rehabilitation protocols. Tasks and stimuli that account for altered temporal processing in affected visual fields may yield better outcomes, consistent with the adaptive approach advocated by [32]. Moreover, the finding that temporal discrimination improves with longer ISIs in the scotoma condition suggests that slowing the presentation rate of sequential stimuli might be beneficial in rehabilitation contexts, a strategy that could be incorporated into the training programs described by [67].

The lack of emotional modulation effects has implications for rehabilitation approaches that leverage emotional content to enhance visual processing. While such approaches have shown promise in other contexts [44] our results suggest that they may have limited effectiveness for improving temporal discrimination in compromised visual fields. Alternative approaches focusing on direct perceptual training, as suggested by [67], may prove more effective for addressing temporal processing deficits.

### Methodological Considerations

A methodological consideration for our paradigm is that it did not include 0 ms inter-stimulus interval (ISI) trials, which would represent a true “one-flash” condition. The inclusion of such trials would allow for a formal analysis of response bias (e.g., using signal detection theory) separately from perceptual sensitivity. However, our approach, which involves measuring the threshold at which two flashes are perceived as one, is a well-established paradigm for assessing the limits of temporal resolution in vision and has been used in numerous previous studies [56].

While a change in response criterion could theoretically influence performance, the data suggest we are measuring a genuine perceptual effect. The impairment in the degraded condition was not a uniform decrease in accuracy across all ISIs (which might suggest a simple bias against responding “two”), but rather a rightward shift of the entire psychometric function. This systematic change, dependent on the temporal separation of the stimuli, is the hallmark of a change in temporal sensitivity. Furthermore, in Experiment 2, emotional primes did not produce any significant main effects or interactions, making it unlikely that a hidden, systematic shift in response bias due to emotion was the primary factor at play.

Another potential factor is the fixed mapping of the response keys (‘z’ for one flash, ‘m’ for two) and the location of the degraded visual field (always on the right). It is possible that a stimulus presented on the right could facilitate a right-handed response (‘m’, for “two”) due to stimulus-response compatibility.

However, this potential confound would predict better or faster “two” responses for stimuli on the right side. Our results show the exact opposite: a profound impairment in performance, with significantly higher thresholds and slower reaction times in the right degraded field. The fact that we observed a strong impairment despite a potential S-R compatibility effect that should have worked against our finding actually strengthens our conclusion. It suggests the negative impact of the signal degradation on temporal processing was so robust that it completely overwhelmed any minor facilitatory effects from S-R compatibility. Nevertheless, future studies could counterbalance response mappings to formally rule out this influence.

Several limitations of the current study should be acknowledged. First, while our scotoma methodology effectively simulated some aspects of visual field defects, it differs from actual scotomas in important ways. The semi-transparent nature of our scotoma (60% opacity) allows some visual information to pass through, unlike absolute scotomas resulting from neural damage. Reference [41] noted similar limitations in their neuroimaging study of artificial scotomas, emphasizing the need for caution when generalizing from simulation studies to clinical populations.

Second, our emotional face manipulation was limited to three basic emotions (angry, happy, neutral) presented as static images. The null effect we observed might not be generalized to dynamic facial expressions or other emotional stimuli, which potentially have more potent effects. As ref. [66] demonstrated that the impact of emotional content on visual processing can vary substantially depending on stimulus characteristics and presentation parameters. Future research could explore whether different types of emotional stimuli might successfully modulate temporal processing.

Affective Pictures: Using complex emotional scenes, such as those from the International Affective Picture System (IAPS), could be a powerful test. Our hypothesis is that these pictures, which often have stronger and more consistent valence and arousal ratings than single faces, might be more effective at engaging emotional processing pathways and influencing the temporal resolution task.

Affective Words: Presenting emotional words (e.g., “war,” “love,” “exam”) as primes could test the influence of top-down, conceptual emotional processing. Our hypothesis is that unlike faces or pictures, which are processed more perceptually, words would rely on semantic networks. Finding a modulation from words but not faces would suggest that this temporal task is susceptible to top-down cognitive influence but not bottom-up perceptual priming from faces.

Several promising directions for future research emerge from our findings. First, investigating the neural correlates of impaired temporal processing under scotoma conditions using EEG or fMRI could elucidate the underlying mechanisms, building on the neuroimaging work of [38]. Second, exploring whether other types of emotional stimuli or different emotional induction methods might modulate temporal processing could clarify the boundary conditions of emotion-perception interactions, extending the research of [43]. Third, examining how individual differences in emotional sensitivity or visual processing abilities relate to performance in this paradigm could reveal important moderating factors, following the approach taken by [9] in their study of temporal processing and reading abilities.

Additionally, future studies could investigate whether perceptual learning or training can improve temporal discrimination under scotoma conditions, potentially informing rehabilitation approaches. The finding that performance in the scotoma condition approached that of normal vision at longer ISIs suggests plasticity in the system that might be leveraged through targeted interventions, as demonstrated by [67] for other aspects of temporal vision.

## 5. Conclusions

In conclusion, our study demonstrates that artificial scotomas significantly impair temporal processing in the two-flash fusion paradigm, with the most pronounced effects occurring at intermediate interstimulus intervals. This finding contributes to our understanding of how visual field defects affect not only spatial but also temporal aspects of vision, extending the work of [42] on the perceptual consequences of artificial scotomas. The absence of emotional modulation effects, contrary to predictions based on [44] research highlights the complexity of emotion-perception interactions and suggests potential limitations to the influence of emotional content on visual processing under challenging perceptual conditions.

These findings have implications for both theoretical models of visual perception, such as those proposed by [46,48], and practical approaches to visual rehabilitation advocated by [68]. By furthering our understanding of how the visual system processes temporal information under compromised conditions, this research takes an important step toward addressing the challenges faced by individuals with visual field defects and developing more effective interventions.

## Figures and Tables

**Table 1:** Accuracy (%) by ISI and Position in Experiment 1.

ISI (ms)	Normal (Left)	Scotoma (Right)	Difference
10	12.8	9.5	3.3
20	20.9	11.8	9.1
30	39.0	12.4	26.6
40	63.0	22.5	40.5
50	80.7	43.8	36.9
60	87.5	68.0	19.5
70	90.6	83.9	6.7
80	89.0	88.3	0.7

**Table 2:** Threshold Analysis for Experiment 1.

Position	Threshold (ms)	95% CI	Slope
Normal (left)	34.78	32.42–37.14	0.096
Scotoma (right)	52.29	50.00–54.59	0.092

**Table 3:** Accuracy (%) by Position and Emotion in Experiment 2.

Position	Angry	Happy	Neutral	Average
Normal (left)	59.4	62.0	60.8	60.7
Scotoma (right)	41.7	41.5	41.8	41.7
Difference	17.7	20.5	19.0	19.0

**Table 4:** Threshold Analysis by Position and Emotion in Experiment 2.

Position	Emotion	Threshold (ms)	95% CI	Slope
Normal (left)	Angry	35.58	33.04–38.10	0.100
	Happy	33.80	31.47–36.12	0.101
	Neutral	33.95	31.40–36.51	0.116
Scotoma (right)	Angry	53.51	50.72–56.31	0.105
	Happy	53.50	50.40–56.61	0.109
	Neutral	52.86	49.84–55.88	0.111

**Table 5:** Reaction Times (ms) by Position and Emotion in Experiment 2.

Position	Angry	Happy	Neutral	Average
Normal (left)	580.0	578.0	578.8	579.0
Scotoma (right)	616.1	615.0	616.0	615.7
Difference	36.1	37.0	37.2	36.7

## Data Availability

Data available in a publicly accessible repository. The dataset for both experiments used in this study are publicly available in https://doi.org/10.17605/OSF.IO/VY4UH.

## Supplementary Materials

The following supporting information can be downloaded at: https://www.mdpi.com/article/10.3390/vision9030079/s1, calculation of differences in luminance between layers.
