# Supplementary material for: A Simulated Visual Field Defect Impairs Temporal Processing: An Effect Not Modulated by Emotional Faces
Source: Vision (Basel). 2025 Sep 16;9(3):79. doi: 10.3390/vision9030079 (PMC12452418; doi:10.3390/vision9030079)
Supplement: Supplementary file 1 [file vision-09-00079-s001.zip › vision-3778823-supplementary.pdf]

Supplementary Material: Luminance Calibration and Contrast Calculations

Display Calibration

All experiments were conducted on a 2560 × 1440 px LCD monitor (100 Hz refresh rate). The display was gamma-linearized via a custom lookup table (LUT), ensuring a linear mapping between digital RGB values and measured luminance. Calibration was performed with a photometer at the centre of the screen.

We defined the monitor's maximum luminance at full white [255,255,255] as:

$$L_{\text{white}} = 150 \text{ cd/m}^2$$

Following linearization, pixel luminance was computed as:

$$L(\text{RGB}) = L_{\text{white}} \times \frac{\text{RGB}}{255}$$

Background Luminance

For the homogeneous medium Gray background [128,128,128]:

$$L_{\text{bg}} = 150 \times \frac{128}{255} \approx 75.3 \text{ cd/m}^2$$

This value served as the baseline mean luminance for both normal and scotoma conditions.

Artificial Scotoma Overlay

The degraded hemifield was generated using a semi-transparent black overlay with opacity $\alpha = 0.60$. Alpha blending was defined as:

$$L_{\text{out}} = (1 - \alpha)\, L_{\text{under}} + \alpha\, L_{\text{black}}$$

With $L_{\text{black}} \approx 0$, this simplifies to:

$$L_{\text{out}} \approx 0.40 \times L_{\text{under}}$$

Applying this to the background luminance:

$$L_{\text{bg, scotoma}} = 0.40 \times 75.3 \approx 30.1 \text{ cd/m}^2$$

Thus, the overlay uniformly scaled all underlying luminance values by 0.40 while preserving local contrast.

Gabor Stimuli and Contrast Definition

Gabor patches (2° visual angle, 1 cpd spatial frequency) were rendered at 30% Michelson contrast relative to their immediate background. Michelson contrast was defined as:

$$C = \frac{L_{max} - L_{min}}{L_{max} + L_{min}}$$

luminance $L_0 = L_{\mathrm{bg}}$. The luminance extrema were therefore:

$$L_{max} = L_0(1 + C), \qquad L_{min} = L_0(1 - C)$$

Normal hemifield (no overlay):

$$L_0 = 75.3 \text{ cd/m}^2$$

$$L_{max} \approx 75.3 \times 1.30 \approx 97.9 \text{ cd/m}^2$$

$$L_{min} \approx 75.3 \times 0.70 \approx 52.7 \text{ cd/m}^2$$

Scotoma hemifield (overlay applied):

$$L_{0,\mathrm{scotoma}} \approx 30.1 \text{ cd/m}^2$$

$$L_{max,\mathrm{scotoma}} \approx 39.2 \text{ cd/m}^2, \qquad L_{min,\mathrm{scotoma}} \approx 21.1 \text{ cd/m}^2$$

expected, the overlay preserved the 30% Michelson contrast while reducing all absolute luminance values by a uniform factor.
